# A rare case of sinus of valsalva-right atrial fistula secondary to an abscess perforation from underlying aortic valve endocarditis

**DOI:** 10.1186/1749-8090-9-124

**Published:** 2014-07-14

**Authors:** Elizabeth S John, Joseph Boyer, Bradford Ledzian, Howard Steward, Richard Moro, Hartmuth B Bittner

**Affiliations:** 1 University of Central Florida, College of Medicine, Orlando, FL, USA; 2 Heart and Lung Transplantation and Mechanical Circulatory Support, Orlando, FL, USA

**Keywords:** Aorto-atrial fistula, *Staphylococcus aureus* endocarditis, Prosthesis mismatch, Effective orifice area

## Abstract

Sinus of Valsalva-right atrial fistulas are abnormal connections between the aorta and the right atrium, and present challenging surgical conditions. An extremely rare etiology of aorto-right atrial fistula is infective endocarditis. This case report presents a 21 year old Caucasian female patient who had native aortic valve *Staphylococcus aureus* endocarditis complicated by sinus of Valsalva abscess perforation associated with an acute heart block, an aorto-right atrial fistula, severe heart failure, and cardiogenic shock. She underwent emergent aortic valve replacement and complex sinus of Valsalva fistula pericardial patch reconstruction and repair. This case report further explores the advantages and disadvantages of different valves for different patient populations, and evaluates the patient’s prosthesis mismatch and effective orifice area.

## Background

The etiology of sinus of Valsalva-right atrial fistulas, abnormal connections between the aorta and the right atrium, is multifarious. These fistulas can be congenital [1], caused by dissecting aneurysms and aortic dissections [2,3], and they can result from operations involving the aorta or aortic valve [4-6]. Another extremely rare cause of aorto-right atrial fistula is infective endocarditis [7].

In this case report, we present a 21 year old Caucasian female patient with native aortic valve *Staphylococcus aureus* endocarditis and multiple cerebral intraparenchymal hemorrhages, bacterial meningitis and newly diagnosed Hepatitis C, complicated by sinus of Valsalva abscess perforation associated with acute heart block, aorto-right atrial fistula, severe heart failure, and cardiogenic shock. She underwent emergent aortic valve replacement and complex sinus of Valsalva fistula pericardial patch reconstruction and repair, which is the subject of this report.

## Case presentation

### Patient history

**Figure 1:**
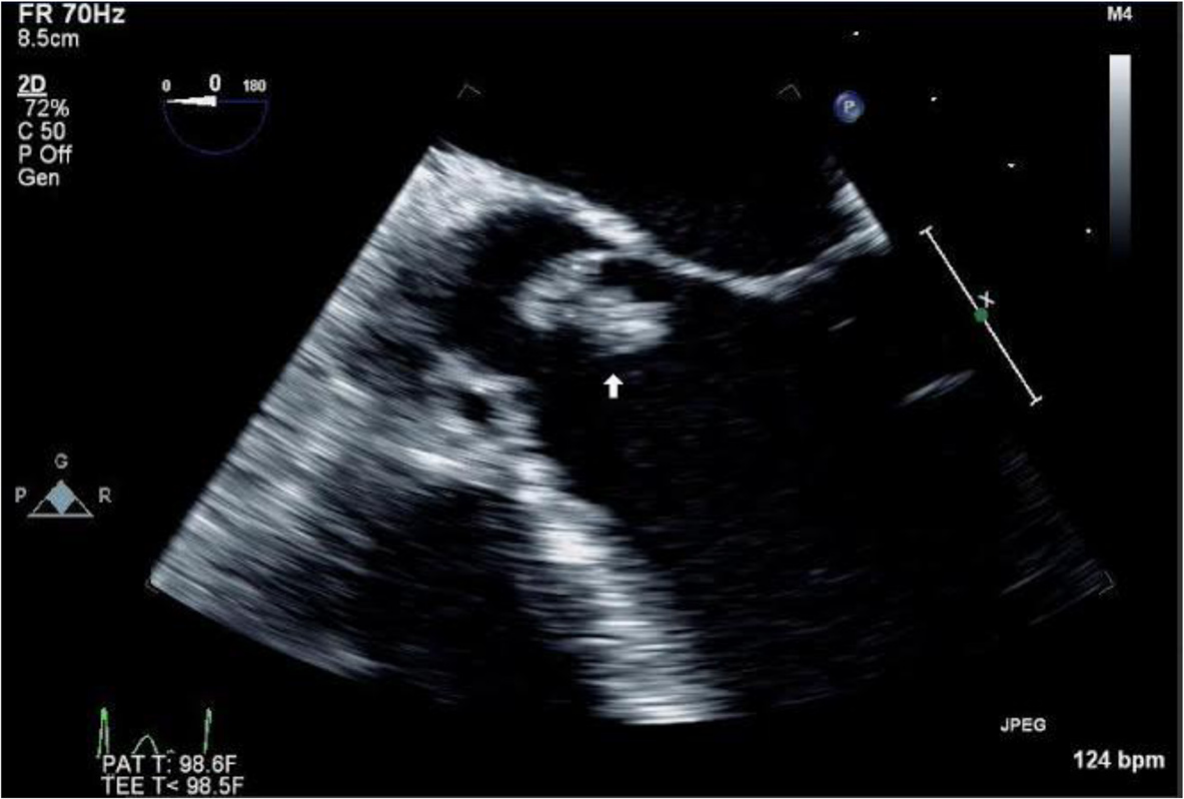
TEE demonstrated a large aortic valve vegetation (at arrow) and severe aortic regurgitation.

A 21 year old intravenous (IV) drug abusing Caucasian female patient was brought into the emergency room by ambulance after being found unresponsive to verbal cues and unable to be awakened. The patient’s past medical history and surgical history were unremarkable, although she had been an IV drug user for 5 years. Upon presentation, the patient was hypotensive, tachycardic, and tachypneic. On physical examination, the patient was not alert or oriented, her pupils were equal, round, and reactive, and she presented with a stiff neck with limited range of motion. Furthermore, she presented with 3+ deep tendon reflexes in both her lower extremities, positive clonus, and an equivocal Babinski sign. The following day, the patient’s blood cultures and lumbar puncture cultures additionally showed a *Staphylococcus aureus* infection.A CT scan delineated multiple small hemorrhages bilaterally and a large 3 cm intraparenchymal bleed in the left frontal lobe. Serologic studies manifested evidence of Hepatitis C, and the patient was severely acidotic as evidenced by her extremely high lactic acid levels. A transesophageal echocardiogram (TEE) demonstrated a 60-65% ejection fraction, a large aortic valve vegetation (2.75 cm × 0.6 cm × 1.16 cm) and severe aortic regurgitation (Figure 1). The patient was started on vancomycin and cefepime while additional studies were acquired.

**Figure 2:**
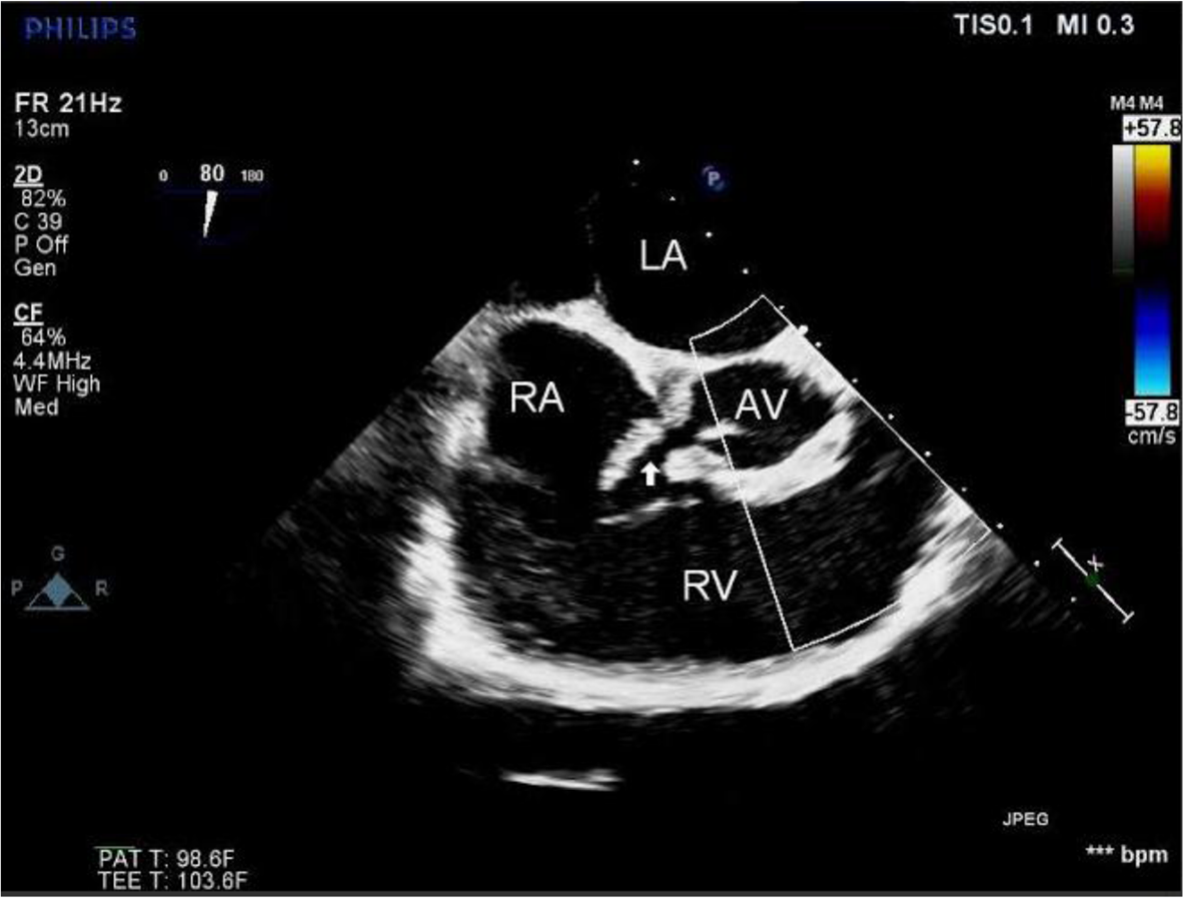
**LA: left atrium, AV: Aortic Valve, RV: Right ventricle, RA: Right atrium; the arrow shows an aorto-RA fistula from the perforation between the aorta and the RA**.

**Figure 3:**
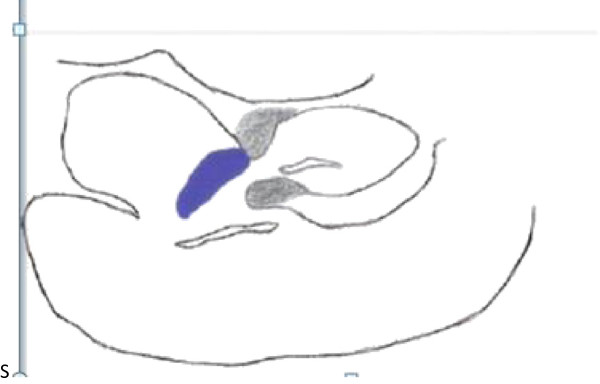
**Blue shaded area: tricuspid regurgitation, gray shaded area: abscess from which the perforation arose; this figure is a cartoon replica of Figure**2**.**

Due to the patient’s recent brain hemorrhage in addition to her current mental status, it was decided that any aortic valve intervention would occur at a later time. The patient began to improve neurologically and was scheduled for an aortic valve procedure. However, the morning before the scheduled surgery, she arrested and was subsequently defibrillated. The patient developed severe heart failure, acute heart block, and pulmonary edema requiring mechanical ventilation. After 8-10 minutes of vigorous cardiopulmonary resuscitation (CPR), the patient was revived and was immediately taken to the operating room.The intra-operative TEE revealed new findings, including aortic insufficiency, a sinus defect of the non-coronary sinus of Valsalva, a rupture of the sinus of Valsalva abscess into the right atrium with fistula formation (Figures 2 and 3), and a new large vegetation at the tricuspid valve. The mitral valve appeared to be intact. A Gerbode ventriculo-atrial defect that permits shunting from the left ventricle to the right atrium was ruled out.

### Surgical procedure

A median sternotomy was performed and the pericardium was quickly opened. The right atrium and left ventricle were decompressed, and after electrically inducing ventricular fibrillation, the ascending aorta was transversely incised distal to the origin of the right coronary artery. A controlled hypothermic cardioplegic arrest was achieved, both cavae were snared with umbilical tapes, and the right atrium was opened parallel to the atrioventricular groove.

Further inspection revealed large vegetations (1.72 cm × 1.16 cm × 1.35 cm) on a defect of the tricuspid annulus and an abscess that perforated through the supra-annular non-coronary sinus area above the tricuspid valve. There was a fistula between the non-coronary sinus, just near the commissure to the right-coronary sinus, and the right atrium just above the annulus of the tricuspid valve. The newly diagnosed tricuspid valve vegetations were a result of the penetration and migration of the original aortic valve endocarditis and vegetations. This area was aggressively debrided to restore healthy tissue.

**Figure 4:**
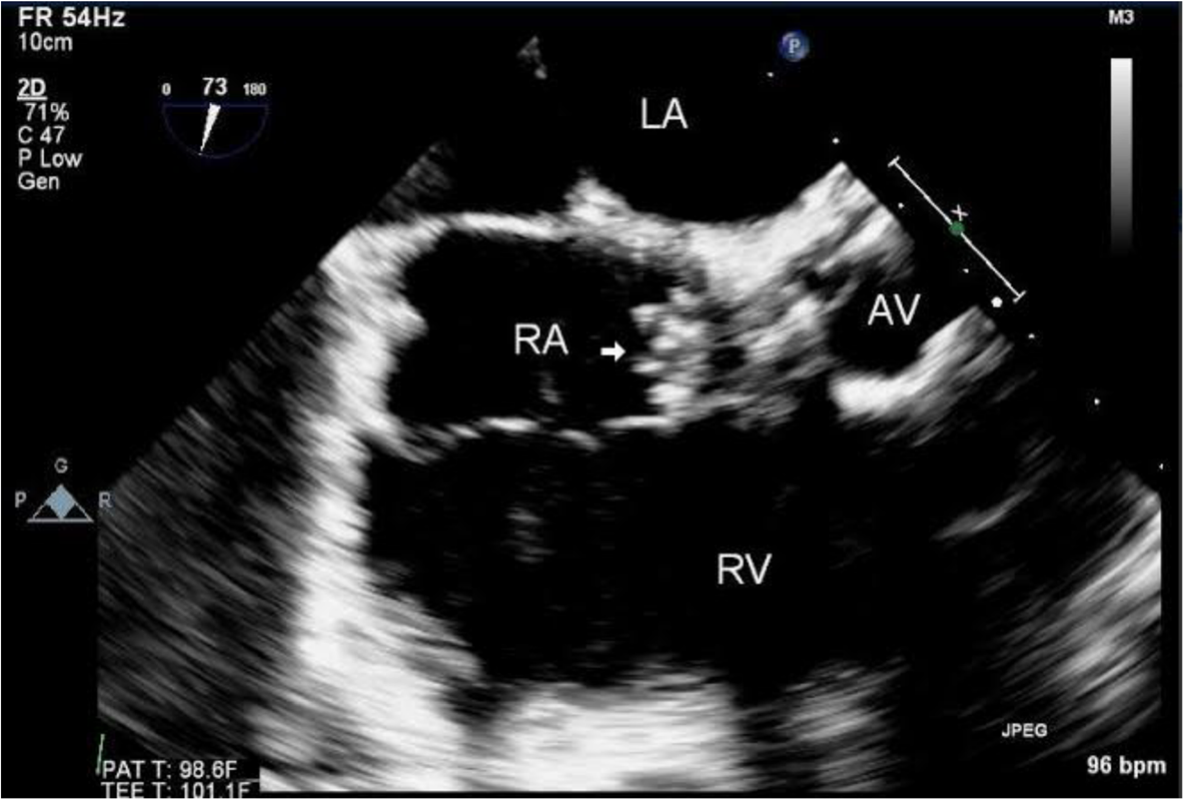
**LA: left atrium, AV: Aortic Valve, RV: Right ventricle, RA: Right atrium, arrow: suture knots and pledgets used for repair.** This TEE after the surgery shows the repair of the aorto-RA fistula.

The autologous pericardium patch was used to close the destructive intra- and peri-annular areas from the atrial side and to similarly close the aortic area of the non-coronary sinus. After repairing the fistula and reconstructing the peri-annular defects and debriding all of the abscess, a diamond–shaped bioprosthetic pericardial patch was placed in a supravalvular position near the superior vena cava, which assisted in a tension-free closure of the near circumferential aortotomy. Because the perforation had inevitably disturbed the conduction system of the heart, to which the patient’s loss of normal sinus rhythm was attributed, atrial and double ventricular epicardial pacing wires were placed for sequential AV pacing.A completion TEE revealed a functioning and competent bioprosthetic aortic valve, no paravalvular leakage, correction of the aorto-right atrial fistula, and an overall well-preserved left and right ventricular function (Figure 4). The patient was transferred to the surgical intensive care unit (SICU) for further monitoring and management. After weaning-off mechanical ventilation and extubation on post-operative day (POD) four and continuation of AV sequential pacing, a permanent dual chamber pacemaker was implanted on POD seven. Almost all of her organ systems recovered fully. Although her neurologic deficit of right sided hemiplegia and motor aphasia attenuated through daily speech therapy and rehabilitation, a transfer to a specialized care facility was required on POD thirty. She was discharged to home on POD forty-one.

## Conclusion

The case of this 21 year old female is particularly interesting for a few reasons. First, infective endocarditis (IE) due to *Staphylococcus aureus* is much more devastating than other IE cases caused by other microorganisms such as *Staphylococcus epidermidis*, *Streptococcus*, and *Enterococcus*. In a prospective study of 194 patients with IE, *S. aureus* IE compared to IE caused by other pathogens was more often associated with severe sepsis (39 versus 6 percent), major neurological events (18 versus 8 percent), multi-organ failure (29 versus 10 percent), and higher mortality (34 versus 10 percent) [8]. In this patient, the *S. aureus* infection was so devastating that it caused annular destruction of the aortic valve, and the development of an abscess that perforated through the aortic-right atrial wall, which precipitated sudden severe heart failure and cardiogenic shock. Finally, the infection caused a large vegetation on a defect of the tricuspid valve.

Another interesting aspect of this case was the manner by which the patient’s aortic valve was fixed. Normally, it is highly recommended to repair a valve instead of completely replacing it because of the increased risk of infection of the prosthetic material with valve replacement. However, in some patients, this is not possible due to the extent of the damage, as was seen in our patient. Another decision that was carefully considered was the type of valve that would be the most beneficial in the repair. It was ultimately determined that a bioprosthetic valve would be more salubrious to the patient as opposed to a mechanical valve. Mechanical valves have been shown to be much more durable and have a lower incidence of primary valve failure compared to bioprosthetic valves in patients under the age of 65 [9]. Studies show the rate of reoperation in patients who receive bioprosthetic valves versus mechanical valves is significantly higher. However, the mortality is the same between the two options [10]. Thus, the ultimate valve choice depends on individual preferences. However, there are contraindications to using mechanical valves, and the valves are highly thrombogenic which mandates lifelong anticoagulation. Our patient, who had many other conditions due to her drug abuse and complicated history, had intracranial hemorrhages that would have been exacerbated by use of an anticoagulant, making a bioprosthetic valve was much more conducive. Bioprosthetic valves have functional properties that are similar to native valves in terms of their similar hemodynamic properties and resistance to thrombosis. In fact, though mechanical valves are more durable, there has been an increase in the number of bioprosthetic valves utilized over the past ten years. Though the reason is difficult to explain, it can be attributed to the unwillingness of many younger patients to be on anticoagulation for the rest of their lives. Moreover, the creation of newer valves have longer expected reoperation-free survivals [11].

Another important factor that was important to consider during the operation was the concern for patient-prothesis mismatch (PPM) using a 19 mm Trifecta aortic tissue valve. PPM is present when the effective orifice area (EOA) of the valve is too small in comparison to body size. This results in hemodynamic instability, as there are higher than expected gradients through normally functioning prosthetic valves. Furthermore, it has been shown to be associated with less regression of ventricular hypertrophy, more cardiac events, and overall a lower survival [12]. For this patient, the mean effective orifice area (EOA) of this particular valve was 1.61 cm^2^. Her BSA was calculated to be 1.58 m^2^. Subsequently, her ratio measured 1.02 cm^2^/m^2^. An EOA of 0.85 cm^2^/m^2^ is generally regarded as the threshold for aortic PPM, thus, this valve was adequate for the patient. Since PPM is a largely preventable cause of failure, it is important to prospectively strategize at the time of operation.

Lastly, this patient’s presentation was of interest because such fistulas between the aorta and a cavity occur rarely in infective endocarditis (IE) patients, and are even more uncommon in drug abusers. In a retrospective multi-center study of 4861 patients with IE, 76 patients were found to have subsequent aorto-cavernous fistulas [1.6%, confident interval (CI) 95%: 1.2-2.0%]. Out of this subject pool, 1.8% of cases were from native valve IE, 3.5% were secondary to prosthetic valve IE, and 0.4% were found in drug abusers [7].

This patient’s case was very complex because of the comorbidities that prevented immediate action to the vegetation, however the case is a springboard through which important factors including EOA, PPM, and valve type had to be considered and important decisions had to be made in such a limited amount of time.

## Consent

Written informed consent was obtained from the patient for publication of this case report and any accompanying images. A copy of the written consent is available for review by the Editor-in-Chief of this journal.

## Abbreviations

ACF: Aorto-cavernous fistulas; CBC: Complete blood count; CPR: Cardiopulmonary resuscitation; CSF: Cerebrospinal fluid; EF: Ejection fraction; EOA: Effective orifice area; IE: Infective endocarditis; IV: Intravenous; POD: Post-operative day; PPM: Patient prosthesis mismatch; RA: Right atrium; TEE: Trans-esophageal echocardiography; SICU: Surgical intensive care unit; WBC: White blood cell.

## Competing interests

The authors declare that they have no competing interests.

## Authors’ contributions

ESJ is the primary author. HB played a pivotal role in manuscript editing, was the senior author and operating surgeon for the case. JB was an operating surgeon for the case, BL and HS were the Physician Assistant Surgeons for the case, and RM produced the images for the paper. All authors read and approved the final manuscript.
